# Commentary: The effect of cochlear implant surgery on vestibular function in adults: A meta-analysis study

**DOI:** 10.3389/fneur.2022.1060370

**Published:** 2022-11-11

**Authors:** Jonas Bruun Kjærsgaard

**Affiliations:** ^1^Balance & Dizziness Centre, Department of Otolaryngology, Head & Neck Surgery and Audiology, Aalborg University Hospital, Aalborg, Denmark; ^2^Department of Clinical Medicine, Aalborg University, Aalborg, Denmark

**Keywords:** cervical vestibular evoked myogenic potential (cVEMP), video head impulse (vHIT), cochlear implant (CI), commentary, meta-analysis

## Introduction

A recent meta-analysis by Vaz et al. explored the vestibular function before and after cochlear implantation (CI) objectivated by cervical vestibular evoked myogenic potentials (cVEMP), the caloric test, head impulse test, and the video head impulse test (vHIT) (1). A specific commentary on their selected literature, data analysis, and the interpretation of cVEMP deserves to be put forward.

## Subsections relevant for the subject

Alterations of Vestibular Function in Cochlear Implantation.

## Discussion

Vaz et al. include data from the paper of both West et al. and Rasmussen et al. even though it is beyond doubt the same patients and preoperative outcomes that are being reported (2, 3). This would magnify the findings and uncertainty originating from a single sample, the opposite of the *raison d'être* of meta-analyses. Furthermore, Vaz et al. report the number of participants in the study of Rasmussen et al. to be 43, while the actual number being reported is 35 (3). This incongruence could perhaps stem from the specific identification number given to the last patient shown in their raw data report, but it is still incomprehensive how this misunderstanding was not corrected when doing the analysis, as Rasmussen et al. stated their sample size in multiple places in both text and figures. These eight extra patients in the meta-analysis were mistakenly added to the “normal” group for the vHIT examination, but for the cVEMP, they were grouped as “abnormal”. This bias would result in an underestimation of the absolute risk for abnormal vHIT and quite strongly overestimate the absolute risk for an abnormal cVEMP result. These mistakes could influence the overall credibility of the study.

Vaz et al. wisely reflect on the reliability of the cVEMP following CI. However, they do not fully encapsulate the complexity of abnormal cVEMP results and by which caution they should be interpreted, despite critical evidence mounting (4, 5). Some important points may be drawn directly from the raw datasets from Rasmussen et al. and Nordfalk et al., which exemplify the challenges of cVEMP usage when evaluating patients before and after CI (3, 6). Rasmussen et al. interestingly found in their repeated measures of the non-implanted ear a change from the first absent to present cVEMP response in 7 out 35 patients (20%), demonstrating in a clinical setting a low grade of repeatability, even for the most extreme outcome of the test. It is obviously debatable to which extend this phenomenon is generalizable, but Rasmussen et al. is not the first study to report such findings (3, 7). The assessment of this phenomenon is hindered as cVEMP results are often only being reported for the implanted side and furthermore only by means of amplitude and latency. This obscures within subject results of uttermost clinical importance, such as how often a previous absent response may be elicited later in the same patient. Nordfalk et al. choose to examine the specific thresholds of cVEMP, which is seldomly seen as an outcome. In this manner, they provide valuable information as they found that amongst the ones with preserved cVEMP after CI the intensity needed to elicit a cVEMP increased in 13 out of 14 subjects (92.8%). This difference in thresholds could perhaps be explained by changes in inner and middle ear mechanics, independent of otolith function as Vaz et al. also briefly mentions (1, 8). If true, the consequence would be to increase the number of absent cVEMP responses non-attributional to otolith damage and thus further challenge its applicability in a clinical setting.

## Author contributions

The author confirms being the sole contributor of this work and has approved it for publication.

## Conflict of interest

The author declares that the research was conducted in the absence of any commercial or financial relationships that could be construed as a potential conflict of interest.

## Publisher's note

All claims expressed in this article are solely those of the authors and do not necessarily represent those of their affiliated organizations, or those of the publisher, the editors and the reviewers. Any product that may be evaluated in this article, or claim that may be made by its manufacturer, is not guaranteed or endorsed by the publisher.
